# Analysis of *Plasmodium falciparum Pfcrt* and *Pfmdr1* genes in parasite isolates from asymptomatic individuals in Southeast Nigeria 11 years after withdrawal of chloroquine

**DOI:** 10.1186/s12936-019-2977-6

**Published:** 2019-10-07

**Authors:** Moses N. Ikegbunam, Charles N. Nkonganyi, Bolaji N. Thomas, Charles O. Esimone, Thirumalaisamy P. Velavan, Olusola Ojurongbe

**Affiliations:** 1 Department of Pharmaceutical Microbiology and Biotechnology, Nnamdi Azikiwe University, Awka, Nigeria; 2 Institute of Tropical Medicine, University of Tübingen, Tübingen, Germany; 3 Department of Biomedical Sciences, College of Health Sciences and Technology, Rochester Institute of Technology, Rochester, NY USA; 4 Faculty of Medicine, Duy Tan University, Da Nang, Vietnam; 5 Department of Medical Microbiology and Parasitology, Ladoke Akintola University of Technology, Osogbo, Osun State Nigeria; 6 Molecular Research Foundation for Students and Scientists, Nnamdi Azikiwe University, Awka, Nigeria

**Keywords:** *Plasmodium falciparum*, Drug resistance, Chloroquine, *Pfmdr1*, *Pfcrt*

## Abstract

**Background:**

A reversal of chloroquine (CQ) resistance following a period of withdrawal has raised the possibility of its re-introduction. This study evaluated the current prevalence of *Pfcrt* and *Pfmdr1* alleles in *Plasmodium falciparum* isolates, 11 years after CQ withdrawal in Southeast Nigeria.

**Methods:**

Filter-paper blood samples were collected from 725 non-febrile individuals, comprising 250 children (≤ 12 years), 250 pregnant women and 225 other adults, between October 2014 and February 2015 in Nnewi town, Southeast Nigeria. Nested PCR followed by direct sequencing was employed for the genotyping of *Pfcrt* and *Pfmdr1* genes.

**Results:**

A total of 103 parasites-positive samples were recovered, comprising of 48 (19.20%) among children, 20 (20.00%) among pregnant women and 35 (15.50%) among other adults cohort. The frequency of the mutant genotype of *Pfcrt* 76T, 75E and 74I was 94.50% each. Parasite isolates from children had a frequency of 100% for mutant alleles in all *Pfcrt* codons while isolates from pregnant women and other adults had a frequency of 91% each in all codons. Haplotype distribution of *pfcrt* gene were 5.45, 0.00 and 76.37% for CVMNK, SVMNT and CVIET, respectively. For *Pfmdr1* gene, the frequency of 86Y, 184F and 1246Y mutant alleles were 8.54, 29.27 and 3.66%, respectively. Amongst the *Pfmdr1* haplotypes analysed, NFD had the highest frequency of 24.4%, followed by YFD at 6.10%. NYF and NYY occurred the least (1.20%).

**Conclusion:**

The high level of *Pfcrt* mutations is suggestive of a sustained CQ pressure on *P. falciparum* isolates in the study area, despite the change of first line treatment from CQ to artemisinin combination therapy for 11 years. A new strategy to ensure the complete withdrawal of CQ from the country is recommended.

## Background

Drug resistance in *Plasmodium falciparum* has led to a major setback in the control and eradication of malaria across the globe, including Nigeria [1, 2]. Chloroquine (CQ), a safe and cheap anti-malarial was once the drug of choice for treating uncomplicated falciparum malaria for several decades [3]. However, the emergence of CQ-resistant malaria parasites, first reported in Southeast Asia along the Thai-Cambodian border during the late 1950s, and now spread across the globe, has greatly hampered the usefulness of this drug [4]. In Nigeria, the first emergence of CQ-resistant malaria parasites was reported in the southern region in 1987 and later spread across the country [1, 5]. Following high level resistance of *P. falciparum* to CQ, Nigeria changed the first-line option for the treatment of malaria from CQ to artemisinin combination therapy (ACT), with artemether-lumefantrine (AL) in 2005 [1, 6]. Published reports have shown that the switch from CQ to ACT in areas where CQ-resistant *P. falciparum* is prevalent has resulted in the re-emergence of CQ-sensitive strains, a few years after the cessation of CQ use [7], although some other studies have reported otherwise [8, 9].

Surveillance of CQ-resistant *P. falciparum* has relied on molecular methods that have allowed for better understanding of the emergence and spread of drug resistance genes. *Plasmodium falciparum* CQ resistance transporter (*Pfcrt*) gene, located on chromosome 7 with 13 exons encoding 424 amino acid transmembrane protein of 48.6 kDa molecular mass, was identified as CQ resistance marker [10]. It is believed that K76T mutation is the principal determinant of CQ resistance and susceptibility [10]. Other common mutations, C72S, M74I, N75E, A220S, Q271E, I356T, R371I, have also been shown to confer resistance in association with K76 mutation [10, 11]. In addition, point mutation in *P. falciparum* multidrug resistance protein 1 (*Pfmdr1*) gene located on chromosome 5, with one exon which codes for P-glycoprotein homologue 1 with 1419 amino acids and 162.25 kDa [12, 13] has been reported to modulate sensitivity and resistance to multiple anti-malarials. It is known that polymorphism, amplification and variation in mRNA expression level of *Pfmdr1* gene are responsible for resistance to various anti-malarials and sudden uptick of multidrug parasites [12]. Mutations at positions N86Y, Y184F, S1034, N1042D, and D1246Y of *Pfmdr1* have been shown to be responsible for either susceptibility or resistance to CQ, quinine, mefloquine, halofantrine, lumefantrine, and artemisinin [14–17].

The prevalence of asymptomatic malaria in Nigeria has been reported to range from 25% [18] to 59% [19, 20] in some regions of the country. Asymptomatic infection is an important obstacle in malaria control since such asymptomatic carriers do not seek treatment and thereby remain reservoirs for the life cycle of the infection to continue. The systematic identification and characterization of *P. falciparum* drug-resistant genes in asymptomatic individuals as part of surveillance intervention strategy is, therefore, very important for reducing the parasite reservoir and transmission of the disease. The current study investigated the prevalence of *Pfcrt* and *Pfmdr1* alleles that have been recognized as CQ markers in *P. falciparum* isolates collected from asymptomatic individuals eleven years after withdrawal of CQ.

## Methods

### Study area, study population and ethics

The study was conducted in Nnewi town, Anambra State, Southeastern Nigeria, with a population of 194,002 people, annual rainfall of about 1.4 m, and lies mainly in the deciduous forest area, spreading towards the grassland belt (National Census, 2006). Malaria is present throughout the year, with a marked increase during the rainy season, which normally runs from April to September.

Filter-paper blood samples were collected from 725 individuals, comprising 250 children under 12 years old and 225 adults during a community survey. Additional 250 filter-paper blood samples were collected from pregnant women attending Nnewi district hospital. All the samples were collected between October 2014 and February 2015. All participants were without malaria symptoms, as defined inclusion criteria accommodated only participants without obvious symptoms of malaria (axillary temperature ≥ 37.5 °C or history of fever 72 h preceeding presentation). Other enrolment criteria included absence of severe illness, a written consent from participants or guardians, and assent in cases where participants were children. Ethical approval for the study was obtained from the Ethics Review Board, University of Nigeria Teaching Hospital (approval number: NHREC/05/01/2008B-FWA00002458-IRB00002323).

### Sample collection, processing and parasite detection

Blood was collected by finger prick and approximately 4 drops of blood were spotted on filter paper, air dried, individually sealed and stored at room temperature until DNA extraction. Genomic DNA was extracted from the dried blood spots using DNeasy Blood & Tissue kit (QIAGEN, Hilden, Germany), according to the manufacturer’s instructions and kept at − 20 °C until use. For the detection and identification of *P. falciparum*, the nested polymerase chain reaction (PCR) method described for all human malaria parasites molecular detection was used [21]. A first-step PCR was performed with specific primers rPLU1 and rPLU5 followed by genus specific (rFAL1/rFAL2—*P. falciparum)*-nested PCR. All PCR reactions were performed in a 25 μl total volume, containing 1X buffer, 2.5 mM MgCl_2_, 200 μM dNTPs, 200 nM primers, and 1U Taq DNA-polymerase (Qiagen, Hilden, Germany).

### Genotyping of *Pfcrt* and *Pfmdr1* polymorphisms

DNA was amplified using nested PCR in a 20 µl reaction volume containing 1× buffer, 2.5 mM MgCl_2_, 200 µM dNTPs, 2.0 mM, Q-solutions (PCR additive), 200 nM primers, 1 U Taq Polymerase and 10 ng of DNA template on a PTC-200 Thermal cycler (MJ Research, USA). The primers, cycling parameters and the amplified codons are listed in Table 1. Amplified PCR products were prepared for sequencing using Exo-SAP-IT (USB, Affymetrix, USA) and directly used as templates for DNA sequencing with the BigDye terminator v. 1.1 cycle sequencing kit (Applied Biosystems, Foster City, USA) on an ABI 3130XL DNA sequencer. Single nucleotide polymorphisms (SNPs) were identified by assembling the sequences with each reference sequence utilizing Sequencher 4.7 software and were reconfirmed visually from their respective electropherograms.

**Table 1:** Primer pairs used to amplify and sequence *Pfcrt and Pfmdr1*

Gene	Primer	Primer sequence	PCR Cycling conditions
*P. falciparum* Genus *P. falciparum*	rPLU1	TCAAAGATTAAGCCATGCAAGTGA	25 cycles of 94 °C/1 min, 58 °C/1 min, and 72 °C/2 min 30 cycles of 94 °C/1 min, 58 °C/1 min, and 72 °C/2 min
	rPLU5 rFAL1 rFAL2	CCTGTTGTTGCCTTAAACTTC TTAAACTGGTTTGGGAAAACCAAATATATT ACACAATGAACTCAATCATGACTACCCGTC	
Pfcrt SNPs Codon 72 and 76	OF P1	5′-GACGAGCGTTATAGAGAATTA-3′	35 cycles of 94 °C/30 s; 56 °C/30 s; and 62 °C/1 min
	OR P2	5′-CCAGTAGTTCTTGTAAGACC-3′	
	NF P3	5′-GGCTCACGTTTAGGTGGA-3′	30 cycles of 94 °C/30 s; 56 °C/30 s; and 65 °C/1 min
	NR P4	5′-TGAATTTCCCTTTTTATTTCCAAA3′	
Pfmdr1 SNPs Codon 86 and 184	OF P5	5′-AGGTTGAAAAAGAGTTGAAC-3′	30 cycles of 94 °C/30 s; 55 °C/30 s; and 65 °C/1 min
	OR P6	5′-ATGACACCACAAACATAAAT-3′	
	NF P7	5′-ACAAAAAGAGTACCGCTGAAT-3′	30 cycles of 94 °C/30 s; 60 °C/30 s; and 65 °C/1 min
	NR P8	5′AAACGCAAGTAATACATAAAGTC3′	
Pfmdr1 SNPs Codon 1034 1042 & 1246	OF P9	5′-GTGTATTTGCTGTAAGAGCT-3′	34 cycles of 94 °C/30 s; 55 °C/60 s and 72 °C/1.5 min
	OR P10	5′GACATATTAAATAACATGGGTTC	
	NF P11	5′CAGATGATGAAATGTTTAAAGATC	29 cycles of 94 °C/30 s; 60 °C/30 s; and 65 °C/1 min
	NR P12	5′-TAAATAACATGGGTTCTTGACT	

## Results

Total of 103 (n = 103) parasite-positive samples were recovered from children (n = 48), pregnant women (n = 20) and other adult cohort (n = 35). The general characteristics of the study population are presented in Table 2. For the children cohort, the mean age of the population was 3.17 ± 0.17 (age range 2 months to 10 years). Gender ratio was 159 male *versus* 92 females. The overall prevalence of *P. falciparum* in the children cohort was 19.20%. For pregnant women, the mean age was 31.10 ± 0.35 (age range: 19 to 59 years) and the prevalence of *P. falciparum* was 8%. In the other adult population, the mean age of the population was 36.83 ± 0.85 (age range: 13 years to 82 years, gender ratio: 0.98 [155:157]). The overall percentage prevalence of *P. falciparum* in this group was 15.60%.

**Table 2:** Demographic characteristics of the population

Characteristics of patients	Children cohort (n = 250)	Pregnant women cohort (250)	Other adult population (n = 225)
Mean age	3.17 ± 0.17	31.10 ± 0.35	36.83 ± 0.85
Age range (years)	< 1–12	19–59	19–82
Gender ratio M:F	159:92	NA	0.98 (155: 157)
Number (%) positive for *P. falciparum*	48 (19.20%)	20 (8.00%)	35 (15.60%)

### Frequency of the *Pfmdr1* mutations

Of the 103 parasite-positive samples, the *Pfmdr1* gene was successfully sequenced in 82 samples. The results for *Pfmdr1* polymorphisms are shown in Table 3. Twenty-one single nucleotide polymorphisms were observed in the *Pfmdr1* gene, with the highest number among pregnant women (15), followed by children cohort (12). The highest frequency of point mutation was observed in the position Y184F (29.3%) with the children cohort recording the highest frequency (38.9%). The frequency of the N86Y mutation was 8.54%, with the children also recording the highest frequency (16.7%). The highest frequency of heterozygote (3.7%) was observed at position Y184F. All the mutations observed were as shown in the Table 3. At codon 1157, 1 (1.22%) sample had synonymous mutation. All the other mutations observed were non-synonymous.

**Table 3:** Frequency and number of *Pfmdr1* mutations in the populations sampled at Nnewi, Southeast Nigeria

Gene	Codon	Children (n = 36)	Pregnant women (n = 20)	Other adults (n = 26)	(Total n = 82)	
		Mutants n (%)	Mutants n (%)	Mutants n (%)	Mutant n (%)	Heterozygote n (%)
Pfmdr1	N86Y	6 (16.67)	1 (5.00)	0 (0.00)	7 (8.54)	0 (0.00)
	Y158H	0 (0.00)	1 (5.00)	0 (0.00)	1 (1.22)	0 (0.00)
	Y184F	14 (38.88)	4 (20.00)	6 (23.08)	24 (29.27)	3 (3.66)
	F74L	1 (2.77)	0 (0.00)	0 (0.00)	1 (1.22)	0 (0.00)
	S164S	1 (2.77)	0 (0.00)	0 (0.00)	1 (1.22)	0 (0.00)
	K1104E	0 (0.00)	1 (5.00)	0 (0.00)	1 (1.22)	0 (0.00)
	Y1004H	0 (0.00)	1 (5.00)	0 (0.00)	1 (1.22)	0 (0.00)
	S1066P	0 (0.00)	0 (0.00)	0 (0.00)	0 (0.00)	1 (1.22)
	T1069T	1 (2.77)	1 (0.00)	1 (3.85)	2 (2.44)	0 (0.00)
	A1077A	1 (2.77)	2 (0.00)	0 (0.00)	1 (1.22)	0 (0.00)
	A1090A	0 (0.00)	0 (0.00)	0 (0.00)	0 (0.00)	1 (1.22)
	N1106S	1 (2.77)	0 (0.00)	0 (0.00)	1 (1.22)	0 (0.00)
	I1117 V	1 (2.77)	1 (0.00)	0 (0.00)	1 (1.22)	0 (0.00)
	R1176G	1 (2.77)	2 (0.00)	0 (0.00)	1 (1.22)	0 (0.00)
	I1122T	1 (2.77)	3 (0.00)	0 (0.00)	1 (1.22)	0 (0.00)
	K1129R	0 (0.00)	1 (5.00)	0 (0.00)	1 (1.22)	0 (0.00)
	S1137S	1 (2.77)	0 (0.00)	0 (0.00)	1 (1.22)	0 (0.00)
	D1246Y	2 (5.55)	1 (5.00)	0 (0.00)	3 (3.66)	0 (0.00)
	G1114G	0 (0.00)	0 (0.00)	0 (0.00)	0 (0.00)	1 (1.22)
	I1143 V	0 (0.00)	0 (0.00)	0 (0.00)	0 (0.00)	1 (1.22)
	T1157T	0 (0.00)	0 (0.00)	1 (3.85)	1 (1.22)	0 (0.00)
	K1154 K	0 (0.00)	1 (5.00)	0 (0.00)	1 (1.22)	0 (0.00)
	T1224T	0 (0.00)	1 (5.00)	0 (0.00)	1 (1.22)	0 (0.00)
	N1243 K	0 (0.00)	0 (0.00)	1 (3.85)	1 (1.22)	0 (0.00)
	R1244G	0 (0.00)	1 (5.00)	0 (0.00)	1 (1.22)	0 (0.00)
Haplotypes						
	NFD n (%)	11 (30.56)	3 (15.00)	6 (23.08)	20 (24.39)	
	NFYn (%)	0 (0.00)	1 (5.00)	0 (0.00)	1 (1.22)	
	YFDn (%)	5 (13.89)	0 (0.00)	0 (0.00)	5 (6.10)	
	NYYn (%)	1 (2.78)	0 (0.00)	0 (0.00)	1 (1.22)	

### Frequency of the *Pfcrt* gene mutations with possible haplotypes

A total of 55 *P. falciparum* samples were successfully sequenced for the *Pfcrt* gene mutation with Table 4 showing the frequency of the mutations at different codons. All the samples had 100% wild type at codon 72. At codons 74, 75 and 76, the frequency of the mutation observed was 94.5% each, with the pregnant women recording 91% each followed by the adult group with 90.5% each. The resultant amino acid changes were all non-synonymous. For the haplotypes analysis, 52 (94.54%) had CVIET, 3 (5.45%) had CVMNK while none of the samples had SVMNT.

**Table 4:** Frequency and number of *Pfcrt* gene mutations with posible haplotypes in Nnewi, Southeast Nigeria

Pfcrt n = 55	Codon	Children n = 23	Pregnant women n = 11	Other adults n = 21	Total n = 55
		Mutant n (%)	Mutant n (%)	Mutant n (%)	Mutant n (%)
Gene	C72S	0 (0.00)	0 (0.00)	0 (0.00)	0 (0.00)
	M74I	23 (100)	10 (90.90)	19 (90.48)	52 (94.54)
	N75E	23 (100)	10 (90.90)	19 (90.48)	52 (94.54)
	K76T	23 (100)	10 (90.90)	19 (90.48)	52 (94.54)
Haplotypes	CVMNK n (%)	0 (0.00)	1 (9.09)	2 (9.52)	3 (5.45)
	SVMNT n (%)	0 (0.00)	0 (0.00)	0 (0.00)	0 (0.00)
	CVIET n (%)	23 (100)	10 (90.90)	19 (90.48)	52 (94.54)

### Frequency of *Pfcrt* and *Pfmdr1* combined mutations

The frequency of *Pfcrt* and *Pfmdr1* combined mutations are shown in Table 5. Combined point mutations of the two most important positions 86Y/76T (8.70%) was observed only in parasites collected from children, while 184F/76T occurred across the three cohorts with frequencies of 39.10%, 27.30% and 23.80% for the children, pregnant women and other adults, respectively. None of the isolates had 1246Y/76T mutations. In total, 30.90% of the parasites analysed harboured 184F/76T combined mutation.

**Table 5:** Frequency of *Pfcrt* and *Pfmdr1* mutations in the three cohorts of asymptomatic individuals in Nnewi district, Southeast Nigeria

Mutations	Children n (%)	Pregnant women n (%)	Other adults n (%)	Total n (%)
86Y/76T	2 (8.70)	0 (0.00)	0 (0.00)	2 (3.60)
184F/76T	9 (39.10)	3 (27.30)	5 (23.80)	17 (30.90)
1246Y/76T	0 (0.00)	0 (0.00)	0 (0.00)	0 (0.00)
86Y/184F/76T	2 (8.70)	0 (0.00)	0 (0.00)	2 (3.60)
86Y/184F/1246Y	0 (0.00)	0 (0.00)	0 (0.00)	0 (0.00)
86Y/184F/1246Y/76T	0 (0.00)	0 (0.00)	0 (0.00)	0 (0.00)

## Discussion

This report provides insight into the *Pfcrt* and *Pfmdr1* genetic profile of *P. falciparum* isolates in asymptomatic individuals in Nnewi, Nigeria, and represent a unique place in the Southeast Nigeria on the prevalence of anti-malarial resistance genes among asymptomatic individuals. Most of the isolates in this study had mutations on *Pfcrt* gene at codons 74I, 75E and 76T, while no mutation was detected at codon 72S. The overall prevalence of *Pfcrt* 76T was 94.56%, which is similar to the prevalence previously reported for isolates in other parts of the country [1, 22, 23]. The present high prevalence of the 76T in *Pfcrt* gene clearly demonstrated that CQ-sensitive strain of *P. falciparum* has not yet re-emerged, 11 years after changing the malaria treatment policy to ACT. This could be attributed to the sustained CQ pressure, possibly facilitated by the availability of CQ in most pharmacies in this location and in many parts of the country [6, 24]. There is also the possibility that there may be persistent circulation of the marker due to drug selection pressure from amodiaquine, and not necessarily from CQ [25]. The persistence of the mutant allele in the present study is in contrast to the report from other African countries such as Malawi [3, 26], Tanzania [27–30], Kenya [30, 31], Mozambique [32], and Ethiopia [7] following CQ withdrawal. In agreement with the current report however, isolates of *P. falciparum* from South America have been reported to harbour mutant alleles, despite the withdrawal of CQ [8]. There has been no study in Nigeria that suggests re-emergence of CQ-sensitive strains after CQ withdrawal, implying that this may require several more years for reversal to be observed or the need to revamp treatment policy in rural areas, or the availability of CQ in local pharmacies. Also, a very similar study conducted in Ethiopia showed continual circulation of *Pfcrt* 76T mutations after withdrawal of CQ for more than 12 years [33].

The present study also showed that the CVIET haplotype was predominant (94.54%) among other haplotypes of *Pfcrt* gene in the study area, which is consistent with other findings in Nigeria [22, 23]. In addition, CVMNK (wild type) haplotypes was detected in only 5.45% of the isolates, mostly from pregnant women and other adult cohorts. Return of CQ-sensitive parasites (CVMNK haplotype) has been reported following cessation of CQ in some malaria-endemic countries, including Malawi [26], Gabon [34] and Brazil [35]. The absence of SVMNT haplotype observed in this study is in line with reports that confirm the absence of this haplotype in West Africa [36].

This study observed that the frequency of *Pfmdr1* gene mutations varied among the isolates from the three cohorts. Codon 184F (29.27%) mutation predominates all other mutations observed in the study area. Confirmed mutations observed had 8.54% and 3.66% for 86Y and 1246Y, respectively, and no mutation was detected for the 1042 and 1246 codon positions. Oladipo et al. observed a higher prevalence in 86Y (62.20%) and 184F (69.00%) in their findings for the *Pfmdr1* gene mutation among pregnant women in Lagos, Nigeria while no mutation was reported at codon 1246 [26]. Mutant *Pfmdr1* N86Y has been linked to amodiaquine pressure in The Gambia [12], Kenya [37] and Nigeria [25]. Thus, the present data could suggest that the parasites in the study area might be resistant to amodiaquine. Studies have also shown that increased copy number of *Pfmdr1* gene increases the risk of failure after treatment with mefloquine [38]. In Nigeria, some authors have reported high prevalence of 86Y allele of *Pfmdr1* in children with acute uncomplicated malaria in some other regions [39, 40].

The present study also reveals double (86Y/76T, 184F/76T) and triple (86Y/184F/76T) mutations in some of the isolates, particularly among children, potentially indicating that CQ exerts a selective pressure in this area. It could also reflect the extensive use of the drugs among children in the area, demonstrating that children are the major reservoirs for these combined resistance markers. Before now, double mutation at positions 184 and 1042 of *Pfmdr1* had been reported to reduce sensitivity to quinine, while mutations at positions 86 and 1042 increases the parasite’s susceptibility to quinine [41, 42]. If this present observation persists, the biggest challenge would be how to treat severe malaria among children, especially those with cerebral malaria, for which quinine is the medication of last resort.

## Conclusion

This study reveals a high prevalence of *Pfcrt* mutant genotypes and haplotypes and low frequency of *Pfmdr1* mutant genotypes, 11 years after the switch in malaria treatment policy from CQ to ACT in Nnewi, Nigeria. This may suggest continual circulation and spread of CQ-resistant *P. falciparum* parasites in the study area. The continued use of unrecommended CQ for the treatment of malaria, and its possible availability in open market in Nigeria needs to be addressed urgently.

## Data Availability

The datasets during and/or analysed during the current study are available from the corresponding author on reasonable request.
